# Mediastinal fat necrosis-an overlooked cause of chest pain

**DOI:** 10.36416/1806-3756/e20240159

**Published:** 2024-08-07

**Authors:** Edson Marchiori, Bruno Hochhegger, Gláucia Zanetti

**Affiliations:** 1. Universidade Federal do Rio de Janeiro, Rio de Janeiro (RJ) Brasil.; 2. University of Florida, Gainesville (FL) USA.

**Figure 1:**
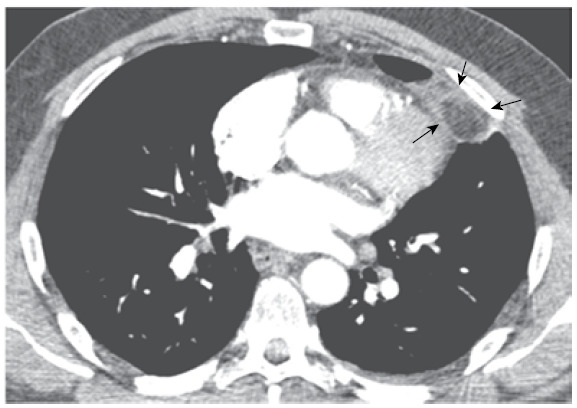
Contrast-enhanced CT image (soft-tissue window) showing an ovoid mediastinal fatty lesion (arrowheads) in the left cardiophrenic region, demarcated by a soft-tissue attenuation ring.

A 55-year-old man was admitted with a three-day history of left anterior chest pain. No fever or another relevant symptom was identified. Physical examination findings were unremarkable. Laboratory tests showed an elevated C-reactive protein level and mild leukocytosis without deviation. Pulmonary thromboembolism was suspected. Chest CT angiography revealed an ovoid mediastinal fatty lesion in the left cardiophrenic region, demarcated by a soft-tissue attenuation ring (Figure 1). In view of these imaging findings, the diagnosis of mediastinal fat necrosis (MFN) was made. A conservative approach was adopted, and the patient’s pain was relieved by nonsteroidal anti-inflammatory drugs.

MFN, also known as epipericardial fat necrosis, is a self-limited cause of chest pain that represents an inflammatory process, usually occurring in the juxtapericardial mediastinal fat and leading to encapsulated fat necrosis. MFN usually manifests with acute pleuritic chest pain in previously healthy individuals and can mimic acute cardiopulmonary processes, such as myocardial infarction, pericarditis, and pulmonary embolism. CT can demonstrate an ovoid or round fatty lesion demarcated by a soft-tissue attenuation rim in the mediastinum. Conservative treatment, such as nonsteroidal anti-inflammatory drug administration, is usually sufficient to relieve symptoms.^1^ ^-^ ^3^

In conclusion, a confident diagnosis of MFN based on imaging findings may help to preclude unnecessary invasive procedures, and conservative symptomatic treatment is the recommended practice.
